# Binding and Fusion of Extracellular Vesicles to the Plasma Membrane of Their Cell Targets

**DOI:** 10.3390/ijms17081296

**Published:** 2016-08-09

**Authors:** Ilaria Prada, Jacopo Meldolesi

**Affiliations:** 1 CNR Institute of Neuroscience, 20133 Milan, Italy; prada.ilaria@yahoo.it; 2 San Raffaele Scientific Institute, DIBIT, via Olgettina 58, 20132 Milan, Italy

**Keywords:** exosomes, ectosomes, extracellular vesicles (EVs), vesicle cargo, multivesicular bodies (MVBs), plasma membrane, retroviral-type membrane fusions, receptors, discharge of luminal cargoes

## Abstract

Exosomes and ectosomes, extracellular vesicles of two types generated by all cells at multivesicular bodies and the plasma membrane, respectively, play critical roles in physiology and pathology. A key mechanism of their function, analogous for both types of vesicles, is the fusion of their membrane to the plasma membrane of specific target cells, followed by discharge to the cytoplasm of their luminal cargo containing proteins, RNAs, and DNA. Here we summarize the present knowledge about the interactions, binding and fusions of vesicles with the cell plasma membrane. The sequence initiates with dynamic interactions, during which vesicles roll over the plasma membrane, followed by the binding of specific membrane proteins to their cell receptors. Membrane binding is then converted rapidly into fusion by mechanisms analogous to those of retroviruses. Specifically, proteins of the extracellular vesicle membranes are structurally rearranged, and their hydrophobic sequences insert into the target cell plasma membrane which undergoes lipid reorganization, protein restructuring and membrane dimpling. Single fusions are not the only process of vesicle/cell interactions. Upon intracellular reassembly of their luminal cargoes, vesicles can be regenerated, released and fused horizontally to other target cells. Fusions of extracellular vesicles are relevant also for specific therapy processes, now intensely investigated.

## 1. Introduction

During the last 15 years, exosomes and ectosomes, the two types of extracellular vesicles (EVs) endowed with physiological and pathological functions, have been among the structures attracting the greatest attention in the scientific community. Exosomes were identified in 2000 as the vesicles trapped within multivesicular bodies (MVBs). Their release to the extracellular space takes place upon the exocytosis of the latter organelles [1]. Considered from the very beginning as vesicles expressed by all types of cells, exosomes started to be recognized and characterized shortly after discovery. Ectosomes, referred to also with other names (shedding vesicles, microparticles, microvesicles and others), are generated at the plasma membrane as small cytoplasmic protrusions covered by specialized membrane rafts which rapidly grow up and are released by fission of their stalk. First described in 1991 in human neutrophils [2], ectosomes have been since investigated separately in various cell types. For years, therefore, little attention was paid to their expression and function in all cells. General interest about these EVs emerged after 2005 and grew progressively over the next years. At present, both exosomes and ectosomes are being intensely investigated, however mostly separate from each other. Time has come, therefore, to consider the properties of the two types of EVs together, focusing in particular on the mechanisms of their generation and function [3].

Numerous aspects of exosome and ectosome generation are known to be different. Among these are the sites of their origin, at the MVBs and the plasma membrane; the length of their intracellular life before discharge, long for exosomes (tens of minutes or more, necessary for their accumulation within MVBs and for their release) and much shorter for ectosomes (a few tens of seconds for their generation at the plasma membrane); their size (diameters of 50–150 and 100–350 nm, respectively); the mechanisms for the accumulation, within their luminal content, of proteins and other macromolecules indicated here as their cargo. In addition, the two types of EVs are often considered molecularly different. Thus, quite a few proteins were proposed as markers of exosomes. Recent studies, however, have shown many such molecules to be present also in ectosomes, although at lower levels. Real markers now recognized, specific of either type of EV, are few. Nevertheless, they remain useful for the identification of the two EVs [3].

Once generated and released, the destinies of the two types of EVs become similar, if not identical. Fractions of both types of EVs undergo dissolution of their membrane. As a consequence, the factors accumulated in their cargo are released to the extracellular space. Most other EVs, however, do not dissolve, but pursue their navigation in the extracellular fluid. In vivo, the navigation of some of them is long-term, sufficient to reach the large fluids of the bodies. Other EVs, however, remain local, establishing interactions with cells defined here as target cells. Such interactions are at least of two types. Some EVs, taken up by various forms of endocytosis, can either be discharged into lysosomes or fuse with endocytic membranes, with ensuing release of their cargo into the cytoplasm. Alternatively, the binding of EVs to target cells is followed by their fusion with the plasma membrane. The different conditions of the two pathways, acidic within endosomes, near neutral in the extracellular space, appear critical for the EV membrane fusion. The process occurring upon uptake of EVs (especially exosomes) into endosomes has been extensively investigated and recently illustrated in comprehensive reviews [4,5]. In contrast, the interaction/binding/fusion of EVs with the plasma membrane, although widely accepted, has never been described in detail. The present mini-review is focused exclusively on the latter process at the surface of target cells. The processes occurring with exosomes and ectosomes appear very similar [3]. Therefore, they will be considered together, under the common definition of EVs.

## 2. Vesicle (EV) Interactions with the Plasma Membrane of Target Cells

As already mentioned, exosomes and ectosomes are generated and released by all types of cells. This property may suggest the two types of EVs to be homogeneous in terms of composition. This is not the case. The two EV types are, in fact, distinct in composition. This can be the case of EVs released from different cells. Even when EVs originate from the same type of cell, some of their properties can be different, depending on reasons such as the state of development of their cells of origin, their functions, and the degree of their stimulation. Large differences have been reported also between wild-type EVs and the analogous EVs of cancer cells [3]. These differences may account for the specificity of EV interactions with target cells. For example, the ectosomes released from platelets are known to interact with macrophages and endothelial cells, but not with neutrophils; the ectosomes from neutrophils to interact not only with platelets but also with macrophages and dendritic cells [6,7]. Analogously, the exosomes released by neuroblastoma cells bind indiscriminately to neurons and glial cells, whereas the exosomes released from stimulated cortical neurons bind only to neurons [8].

**Figure 1:**
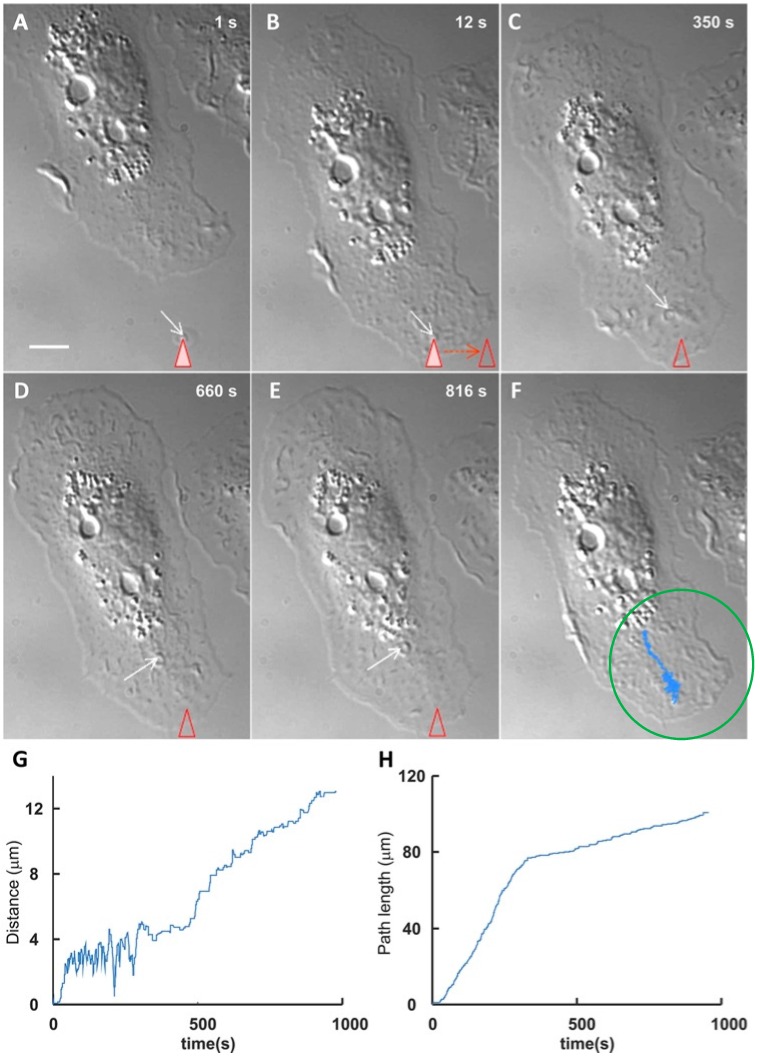
Optical tweezer-induced direct interaction of a vesicle (EV) with the surface of a microglial target cell. The figure illustrates the interaction and binding events occurring in a cultured microglial cell interacting with a single EV (marked by arrows) trapped by optical tweezers (marked by triangles) (**A**) and then transferred near the periphery of the cell surface (**B**); Upon its release, the EV exhibited a number of oscillations (**)F**)), after which it started sliding over the cell surface towards the central area of the cell (**C**,**D**); After reaching a critical site of the cell surface, the EV sliding decreased markedly (**E**); (**F**) reports the whole pathway (blue area) followed by the EV, illustrated by the separate steps of (**C**–**E**). Scale bar in A = 5 µm. The morphological images are illustrated also quantitatively in (**G**) and (**H**). Notice in G the initial oscillations reported also by the cell in (**F**); in (**H**) the strong reduction of the speed, evident after approximately 400 s. Such a reduction is probably due to the receptor binding that anticipates the EV/cell fusion, occurring at the surface or upon internalization into endosomes. Modified with permission from Reference [10].

In order to clarify in detail the properties of the EV-cell interactions, the investigation has been expanded using new approaches by which the temporality and the dynamics of the process can be established [9]. Excellent results have been obtained by employing optical tweezers. By this approach, a single EV of known nature and origin is captured and transferred to the surface of various cell types in primary culture. Interestingly, sliding results can be different. In microglial cells, EVs from astrocytes were found to exhibit directional, long movements, whereas in astrocytes they exhibited only minor oscillations close to the site of first adhesion. After some duration of surface sliding, the EVs were seen to markedly reduce their movement (Figure 1), establishing a binding that soon evolved into fusion [10].

In view of the specificities summarized above, the variable properties of the EV-cell interactions can be attributed to the genes expressed by the original and target cells. Specifically, the interactions appear due to the cell surface proteins necessary for EV binding and compatible for the ensuing fusion to get started.

## 3. EV Binding to the Plasma Membrane of Target Cells

Binding and fusion of EVs to the external surface of their target cells should be first distinguished from the apparently analogous fusion processes taking place inside the cell, during organelle traffic, exocytosis, endocytosis and other events. Intracellular binding and fusion require the involvement of various proteins, including actin, other cytoskeletal proteins and numerous forms of soluble *N*-ethylmaleimide-sensitive protein receptor (SNARE), which have nothing to do with the surface binding and fusion processes. Among the latter processes, the ones previously intensely investigated are virus fusions, which occur with the participation of four classes of proteins [11]. Proteins of class I and II have been identified as possibly involved also in the binding and fusion of EVs. Here we will briefly consider the possible involvement of class I proteins in the EV-cell binding processes. The ensuing fusion processes will be considered in the next section.

The initial interactions of EVs are expected to require specific, high affinity binding of at least two surface proteins, one protruding from the EVs, the other from the plasma membrane of the target cells. The surface proteins of class I, syncytin-1 and syncytin-2, were discovered on placental trophoblast plasma membrane, where they participate in the cell-to-cell fusion process [12,13]. These proteins are not limited to the plasma membrane of placental cells. They have been found also on human gametes [14], blood cells [13], osteoclasts [15], differentiating myoblasts [16], pituitary gland cells and various types of tumor cells [17,18]. Moreover, they are exposed by trophoblast exosomes [13] and may be present also on the EVs of other cell types.

**Figure 2:**
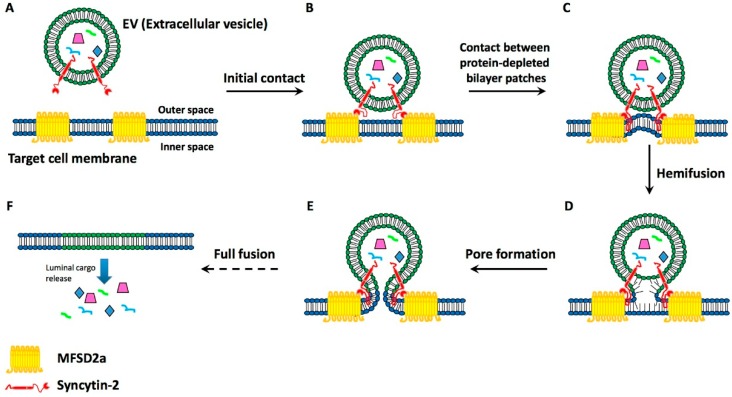
Illustration of the binding and ensuing fusion of an EV to the plasma membrane of its target cell. The EVs (such as the one shown in Figure 1) include, at their surface, domains of the trans-membrane proteins syncytins. (**A**) An EV with syncytin-2 is approaching a target cell which, in the plasma membrane, exhibits the syncytin 2-specific receptor, HFSD2a; (**B**) The EV protein and its receptor appear bound to each other; Hydrophobic loops of the vesicle protein begin to deepen into the plasma membrane, contributing to its molecular re-arrangement, with protein depletion of its external layer (**C**); This is followed by the hemifusion of the EV membrane with the cell target plasma membrane (**D**); followed by the re-organization of the two, closely attached membranes, with their dissolution at the fusion site (**E**); The fusion induced the ensuing insertion of the EV membrane in the target plasma membrane, and the release to the cytoplasm of the luminal cargo molecules: proteins, RNAs and small DNA sequences (**F**).

The interest in these proteins was greatly increased by the identification of two high affinity binding proteins, the receptors Major Facilitator Superfamily Domain 2a (MFSD2a) and Soluble Carrier Family 1 (ASCT-2). These proteins belong to families of carbohydrate and neutral amino acid transporters, respectively [14,19,20]. Properties of the syncytin-2/MFSD2a binding were altered by some, but not all, single-nucleotide mutations of the syncytin gene, and by *N*-glycosylations of the protein [21]. Interestingly, syncytin-2 and MFSD2a were found to exhibit a critical distribution in the human gametes destined to fuse [14]. Moreover, the syncytin-1-equipped exosomes from placental trophoblasts were shown to bind and fuse with blood cells [13]. Summing up, syncytins are known proteins possibly involved in the binding of EVs to target cells, i.e., in the process that precedes fusion (Figure 2).

## 4. Present Knowledge about EV-Target Cell Surface Fusions

The knowledge about EV/target cell fusion (Figure 2), analogous to the fusions taking place between the plasma membranes of two cells, is based on two types of information, the first from the fusions of viruses with cells [11], the other from the data about the binding processes reported in the preceding section [12,13,14,15,16,17,18,19,20,21]. In order for fusions to take place, several events need to take place, including the insertion, in the target cell plasma membrane, of the hydrophobic segments of fusogenic proteins, followed by lipid reorganization, protein restructuring and membrane dimpling [11]. Considered from the viral point of view, the two proteins involved in the fusion of at least some EVs belong to the classes I and II. Class I proteins, syncytin-1 and syncytin-2, are composed of α-helix-rich pre-fusion trimers that insert their hydrophobic fusion peptides in the target membranes. The two proteins then refold as post-fusion trimers. Class II includes the Epithelial Fusion Failure 1 (EFF-1) protein [22]. This protein, not considered here for binding because its possible receptor is unknown, includes β-sheet-rich pre-fusion homodimers and heterodimers that include loops destined to be inserted in the target membrane. At the end of the process the dimers refold into post-fusion trimers [11].

During the time between the pre-folding and post-folding of their fusogenic proteins, the EV and cell plasma membranes become continuous (Figure 2). This process is critical because, upon their discharge into the cytosol, it makes the molecules of the luminal cargo of EVs start carrying out their functions. Bioactive macromolecules of the cargo include proteins, various mRNAs, several miRNAs, and some DNA sequences. Within the cytoplasm the environment is crowded. The diffusion rate of the cargo macromolecules is variable, and their effects occur at different times. Among the discharged proteins, some are common to many eukaryotic cells, others to few cells only. Additional proteins are synthesized according to the mRNA and the DNA specificity. The relevance of protein functions is variable, from ordinary to unique. Highly important are the miRNAs that contribute significantly to the turnover of many proteins, thus inducing effects that may be stimulatory or inhibitory for target cells [23,24]. Additional consequences of the fusion, dependent on the properties of the donor and recipient cells, include changes of the gene expression. Taken together, the processes triggered by the macromolecules released by the EVs lead to the reprogramming of the target cell structure and function.

EV transfer from the cell of origin to a target cell does not necessarily account for a complete interaction process. In target cells, in fact, the cargo and membrane molecules transferred upon EV fusion can accumulate again into vesicles that are released and then fuse to other target cells. This horizontal transfer, taking place among the cells of a tissue, could be expanded also to other cell types, contributing to many physiological processes such as coagulation, stem cell renewal and expansion, and also to the pathogenesis of diseases including inflammation diseases (atherosclerosis, angiogenesis and others), diabetes, and the growth, invasion and metastases of tumors [5,23,24,25,26,27]. Based on the analysis of EVs accumulated in urine, blood sera, saliva or cerebrospinal fluids, the molecules released by patients can also be employed for their diagnosis and prognosis [5,27,28,29,30]. Finally, manipulated vesicles can be employed in new prospective therapies. This issue is discussed in the following section.

## 5. Perspectives of Therapy

Knowledge about their origin and properties stimulated the idea of employing EVs for therapy [5]. A problem that was initially considered was the nature of the vesicles to be employed for the task. Stem cell-derived EVs, loaded with exogenous genetic cargoes, appeared naturally equipped for human genetic therapies [31,32]. This approach, however, has remained without adequate development. More recently, the interest switched towards the delivery of drugs. For this purpose a few technical problems were considered, such as the need for EV stabilization, for example by glycosylation of their surface peptides [33,34]. The present perspectives are based on the design of exogenous EVs, loaded with clearly defined therapeutic cargoes and appropriately engineered with surface markers, to assure their targeting to diseased cells. These vesicles are expected to become useful for the development of a drug nano-delivery system, appropriate for future targeting to specific tissues, such as the human brain [28,35].

## 6. Conclusions

The task of the present review was the demonstration that specific binding and fusion of EVs with the plasma membrane of target cells is a process of great interest for cell physiology and pathology. Binding and fusion of EVs does occur also upon their uptake by the endocytic system, extensively presented in [4,5]. However, not only the timing but also the processing of the internalized EVs could be different from that of EVs undergoing direct fusion with the plasma membrane.

The EVs are often defined as structures that have displaced the external borders of cells, from the plasma membrane to the limit of their navigation. The findings reported here have documented additional properties of EVs including, among others, their horizontal intercellular transfer of macromolecules; their target cell signaling induced by the intercellular traffic of macromolecules; their long-term control of gene expression. The identification of these and other extraordinary processes has introduced new concepts and ideas, not only in cell biology and physiology, but also in medicine. At present, in fact, the role of EVs is being evaluated also by procedures employed for the diagnosis, prognosis, evaluation and therapy of single patients. Based on the results of their intense, ongoing investigation, the EVs can therefore be envisaged as new tools, employed for the progress of biomedical sciences, destined to be further expanded in the next few years.
